# Symptom variation up to two years post-SARS-CoV-2 infection in Children and Young People: results from the Children and young people with Long Covid (CLoCk) study

**DOI:** 10.1186/s12879-026-13951-x

**Published:** 2026-07-11

**Authors:** Natalia K. Rojas, Roz Shafran, Terence Stephenson, Alvin Richards-Belle, Esther Ortega-Martin, Emma Dalrymple, Isobel Heyman, Fiona Newlands, Kelsey McOwat, Ruth Simmons, Snehal M. Pinto Pereira

**Affiliations:** 1 Present Address: Division of Surgery & Interventional Science, Faculty of Medical Sciences, University College London, London, WC1E 6BT UK; 2 UCL Great Ormond Street Institute of Child Health, 30 Guilford Street, London, WC1N 1EH UK; 3 UCL Division of Psychiatry, Maple House, 149 Tottenham Court Road, London, W1T 7NF UK; 4 Department of General Economy (Sociology), Faculty of Nursing and Physiotherapy, University of Cadiz, Cadiz, 11009 Spain; 5 Immunisation Department, UK Health Security Agency, 61 Colindale Avenue, London, NW9 5EQ UK

**Keywords:** COVID-19, Post Covid-19 condition, Long COVID, Cohort study, Mental health, Children and Young People

## Abstract

**Background:**

Post Covid-19 Condition (PCC) can fluctuate over time, yet, no in-depth investigation of the heterogeneous PCC trajectories that can exist in children and young people (CYP) has been undertaken. We aim to examine associations between PCC trajectories in CYP over 2-years following infection and (i) factors prior to the COVID-19 pandemic/infection including socio-demographic variables (e.g., age, sex, ethnicity), health and educational needs status and (ii) factors subsequent to infection including the nature, number, functional impact and severity of symptoms, as well as mental health and wellbeing.

**Methods:**

943 PCR-test positive CYP (enrolled January-March 2021) were followed-up over two-years (till January-March 2023). Five PCC trajectory groups were specified: (i) chronic, (ii) recovered, (iii) fluctuating, (iv) late onset and (v) never PCC. These groups were compared in terms of factors at baseline using multinomial logistic regression and concurrent health during the two-year period using Chi-Square/Fisher’s Exact tests.

**Results:**

Baseline factors prior to the pandemic/infection such as female sex, older age, poorer pre-infection mental and physical health, prior healthcare use, and educational needs were strongly and consistently associated with adverse PCC trajectories. Compared to those aged 11-to-14-years at infection, those aged 15-to-17-years had a 2.44 (95%CI:1.39,4.26) higher risk of being in the chronic group (compared to the never group). Similarly, the risk of being in the fluctuating group was 1.57 (95%CI:1.04,2.37), the recovered group was 2.08 (95%CI:1.10,3.92) and the late-onset group was 1.50 (95%CI:1.08,2.07). Other sociodemographic factors, such as ethnicity and region of residence, had more modest and inconsistent associations. PCC trajectories differed by concurrent number, frequency, functional impact and severity of symptoms and mental health. CYP with chronic PCC consistently reported a higher median number of symptoms (5+) compared to the other groups (median symptoms ≤ 3). Mental health and wellbeing, of the chronic PCC group was also consistently worse (e.g., 41% of the chronic group consistently were classified as ‘cases’ on the Strengths and Difficulties scale vs 17%-to-2% of the other groups).

**Conclusions:**

There were consistent differences between PCC trajectories, in terms of sex, age, pre-infection mental and physical health, healthcare use, and educational needs. Understanding factors associated with PCC trajectory heterogeneity in CYP and how these trajectories differ over time can help with treatment planning.

**Supplementary Information:**

The online version contains supplementary material available at https://doi.org/10.1186/s12879-026-13951-x.

## Introduction

The COVID-19 pandemic has had an unprecedented impact on children and young people (CYP) in numerous ways including school closures, infection and loss of loved ones [1]. One lasting legacy of the pandemic, is the longer-term impact of infection, commonly known as ‘Long Covid’ or Post COVID-19 Condition (PCC). PCC in CYP is defined as presenting with one or more impairing, persisting, physical symptom(s) that may fluctuate or relapse, either continuing or developing post-SARS-CoV-2 infection, and arising (i) within three months and lasting two months [2], or (ii) lasting three months or more [3] after initial infection.

It is difficult to definitively establish the prevalence of PCC in CYP, due to inconsistencies across/lack of use of definitions [4, 5], but systematic reviews report prevalence of persisting symptoms ranging from 4%-to-66% [6–8]. The reviews identify over 200 symptoms associated with PCC in CYP with headaches, fatigue, and shortness of breath among the most prevalent [6–8]. Some symptoms appear to be directly attributable to SARS-CoV-2 infection (e.g., loss of taste and smell [9]), whereas others are more general (e.g., anxiety, low mood and headache). Symptoms are often considered in isolation. For example, some research focuses on biological aspects of PCC [10] while others focus on the mental health impact of PCC [11, 12]. However, symptoms such as fatigue could be viewed as both a physical and mental health symptom [13]. Furthermore, within an individual, mental and physical health are inextricably linked with poor mental health affecting physical health and vice versa [14–18]. Therefore considering ‘symptoms’ without the physical/mental health dichotomy warrants consideration.

The CLoCk study is the largest longitudinal cohort study of PCC in CYP, including over 30,000 participants from across England [19]. It has demonstrated that symptoms commonly reported during acute COVID-19 infection are similar to those reported at 3-, 6-, 12- and even 24-months post-infection, with fatigue and shortness of breath being particularly prominent [20]. The CLoCk study also showed that symptoms fluctuate over time and that some CYP report symptoms for the first time at 6- and 12-months post-infection [21]. Thus, there is notable variation in symptoms (and PCC) over time. For example, 70% of CYP who met the World Health Organization (WHO) clinical case definition of PCC with symptoms occurring within 3-months of infection recovered within 2 years [20]. Furthermore, 7% of those who met the PCC definition at 3-months continued to have PCC persistently at 6-, 12- and 24-months post-infection [20]. Yet, to date, little attention has been given to the waxing and waning of PCC symptoms. This is an important research gap because ignoring the potential for PCC to relapse and remit could lead to misclassifying CYP as having recovered from PCC when, in reality, their PCC is ‘fluctuating’.

The above-described variation in CYP’s PCC trajectory raises several important questions. First, there are questions around the different groups of CYP that have different PCC trajectories. For example, do the CYP who recover from PCC have similar characteristics to those who experience a fluctuating course of their illness, and/or those who continue to experience debilitating symptoms over two years post-infection? Second, there are questions regarding the nature of PCC post-infection. For example, is it the case that CYP meet PCC definitions due to having one continuous symptom over time (such as fatigue) or do they meet the definition but the specific symptom itself changes over time (e.g., initially fatigue but then evolving to headaches)? Additionally, how many symptoms do CYP with PCC experience and does this change over time? Understanding which groups of CYP are more likely to have different PCC trajectories and the symptom profiles of the different PCC trajectories would help with establishing the natural course of PCC and possible subgroups and symptoms to target for intervention.

Our overarching aim is to describe the symptom profiles of CYP with differing PCC trajectories over a two-year period post-SARS-CoV-2 infection.

Specifically, we will examine associations between PCC trajectory membership and:

(i) Factors prior to the COVID-19 pandemic/infection including socio-demographic variables (e.g., age, sex, ethnicity), health and educational needs status.
(ii) Factors subsequent to infection including the nature, number, functional impact and severity of symptoms, as well as mental health and wellbeing over the 2-year period following infection.

## Methods

**Fig. 1:**
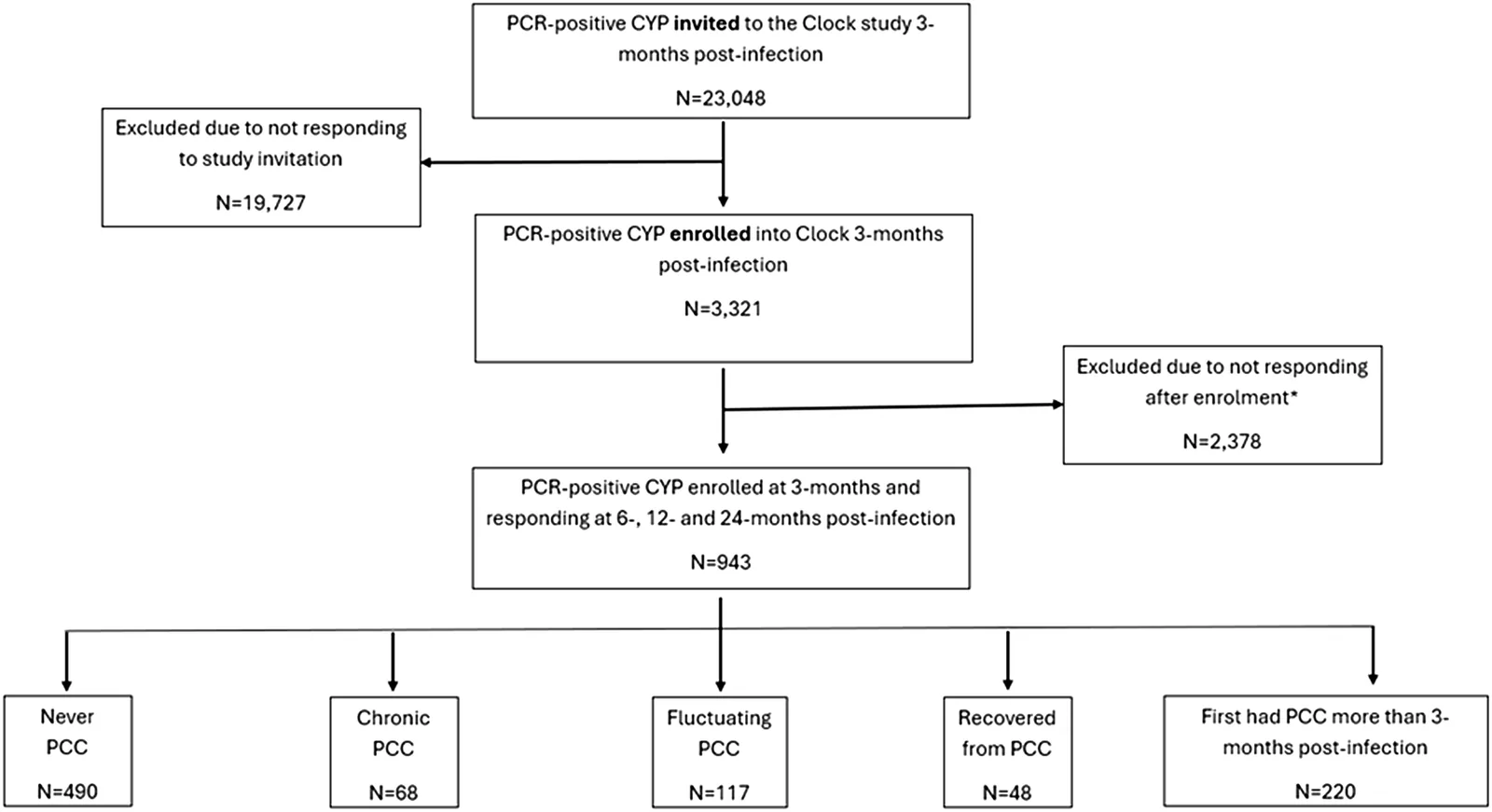
Flow of CYP in the CLoCk study, from invitation at 3-months post-PCR-proven SARS-CoV-2 infection to analytical sample under consideration (*N* = 943). Note: CYP were assessed in terms of meeting/not meeting the Delphi definition of PCC [3] at eachtime point as follows: At least one symptom on the composite questionnaire and at least some or a lot of problems with mobility, self-care, doing usual activities, having pain or discomfort, or feeling very worried, sad or unhappy (see "Measures" for details). *Excluded after enrolling 3-months post-infection and due to non-response at 6-, and/or 12- and/or 24-months post-infection

CYP undergoing SARS-CoV-2 PCR-testing between September 2020 and March 2021 were identified through the UK Health Security Agency’s (UKHSA) database and invited to participate in the Children and young people with Long Covid (CLoCk) study [19, 22]. In brief, PCR-test positive CYP were matched to test-negative CYP on key demographics (age, sex, region of residence, month of test) at study invitation; those providing informed consent completed online questionnaires 3-, 6-, 12- and 24-months post-testing (the number of data collection sweeps varied depending on the CYP’s month of PCR-testing) [19]. Data collection started on April 1st, 2021, and was finalised on May 1st, 2023 [19]. To best align WHO [2] and Delphi [3] definitions of PCC, this manuscript defines clear inclusion and exclusion criteria. Inclusion criteria compromised CYP who PCR-tested positive for SARS-CoV-2 and were enrolled into the CLoCk study 3-months post-infection, with follow-up questionnaire response at 6-, 12- and 24-months post-infection (*N* = 943; Fig. 1). Exclusion criteria included CYP without PCR-confirmed infection at study invitation, those not enrolled at the 3-month timepoint, and those who did not provide follow-up data at one or more subsequent timepoints.

### Measures

UKHSA provided data on age at PCR-testing (grouped here as: 11–14, 15–17 years), sex at birth and region of residence. Index of Multiple Deprivation (IMD) was derived from CYP’s lower super output area, that is, small local area level-based geographic hierarchy, and used as a proxy for socioeconomic status (grouped into 5 quintiles from most to least deprived) [23].

#### Composite questionnaire

The online questionnaire CYP filled in (at each follow-up) is described in detail elsewhere [19] (and briefly in Table S1, see Additional File 1). The questionnaire included elements of the International Severe Acute Respiratory and emerging Infection Consortium (ISARIC) Paediatric COVID-19 follow-up questionnaire [24] and recent Mental Health of CYP in England surveys [25].

At study enrolment (3-month post-infection), the questionnaire included items to assess:

- Ethnicity
- Previous Health: Including previous (i.e., prior to COVID-19 test) health and medical treatment (including, e.g., accessing talking therapy and being prescribed a medication (e.g., to help manage concentration) by a doctor)
- Previous Educational Status: Presence of an Education, Health and Care Plan (EHCP), which identifies CYP’s special educational, health and social needs and outlines the additional support required to meet these needs [26], and can act as proxy for neurodiversity (though not all neurodiverse CYP may have an EHCP). Presence of Learning difficulties prior to the COVID-19 pandemic was also assessed

At all four time-points, the questionnaire included items to assess current health, quality of life and wellbeing including:

- Symptoms: CYP were provided with a list of 21 symptoms to choose from, including an option of ‘other’ (at 12- and 24-months, the extra symptom: ‘sleep problems’ was added to the list [19])
- Functional impact and severity of symptoms: CYP were asked whether they had (i) contacted their doctor about their COVID-19, (ii) attended a hospital for their COVID-19, and (iii) been hospitalised overnight due to their COVID-19. At the 12- and 24-month data collection sweeps CYP were asked to rate the severity of their symptoms and impact on their functioning (on a scale of 0-100, with 0 denoting “not severe at all/no impact” and 100 denoting “extremely severe/extreme impact”)
- Strengths and Difficulties Questionnaire [27]: which measures emotional and behavioural difficulties. It consists of 25 items measuring overall problems, their impact, as well as looking specifically at emotional, conduct, hyperactivity, peer relationships and prosocial behaviour. The SDQ scale has been shown to have satisfactory internal consistency (α = 0.73) [27]
- Short Warwick and Edinburgh Mental Wellbeing Scale [28]: measures mental wellbeing, with items relating more to functioning as opposed to feelings. It has been validated in CYP aged 15–21 and has high internal consistency (α = 0.78 to α = 0.88) across several populations including the UK general population [29–32]
- EQ-5D-Y [33]: measures problems (none/some/a lot) in 5 domains: mobility, self-care, doing usual activities, having pain/discomfort, and feeling worried/sad/unhappy [33]. The scale has been shown to be feasible, reliable and valid, with, for example, percentages of agreement in test–retest reliability ranging from 69.8-to-99.7% [34]. This scale was used in the operationalisation of PCC (described below)

#### Operationalising PCC

Aligning the WHO [2] and Delphi [3] definitions of PCC, we limit our analysis to PCR-positive CYP for whom we have data 3-months post-infection. CYP were classified as meeting/not meeting the research definition of PCC [3] at each time point as follows:

At least one symptom from the pre-defined list (described above) and at least some or a lot of problems with mobility, self-care, doing usual activities, having pain/discomfort, or feeling very worried/sad/unhappy (as measured on the EQ-5D-Y scale).

Once PCC at each time-point was determined, we classified CYP into one of five PCC trajectories:

(i) Chronic PCC (i.e., CYP persistently meeting PCC definition at 3-, 6-, 12- and 24-months post-infection).
(ii) Recovered from PCC (i.e., only meeting PCC definition 3-months post-infection).
(iii) Fluctuating PCC (i.e., met PCC definition at 3-months with subsequent non-persistent relapses).
(iv) First had PCC more than 3-months post-infection (i.e., late onset with CYP first meeting PCC definition at any timepoint after 3-months either once, twice or thrice).
(v) Never had PCC.

### Analysis

Analyses were conducted using Stata v17 [35]. Associations between pre-pandemic/infection variables (i.e., independent variables: socio-demographic characteristics, health factors and educational needs) and PCC trajectory membership were examined using separate multinomial logistic regression models for each independent variable. The dependent (outcome) variable in these models was PCC trajectory membership, with “Never PCC” as the reference category. Results are presented as Relative Risk Ratios (RRRs). Associations between factors subsequent to infection (i.e., the nature and number of symptoms, mental health and wellbeing) and the concurrent PCC trajectories were examined using Chi-Square or Fisher’s Exact tests as appropriate. To assess whether there were any differences between (i) severity and (ii) functional impact and concurrent PCC trajectories, we used Kruskal-Wallis tests due to the non-normal distribution of the data.

Details on all variables of interest examined are summarised in Table S1 (Additional File 1). 

## Results

23,048 PCR-positive CYP were invited to the CLoCk study when they were 3-month post-infection. Of these, 3,321 enrolled and 943 continued to provide data at 6-, 12-, and 24-months post-infection (Fig. 1). CYP in the analytical sample were broadly similar to those invited to the CLoCk study, with some differences. For example, those in the analytical sample were more likely to be female (68.4% vs. 53.9%); older (57.5% were 15-to-17-years-old vs. 53.8%) and from the least deprived IMD quintile (23.8% vs. 14.2%; Table 1). In the analytical sample, questionnaires were returned at a median of 3.4 (interquartile range (IQR): 3.0-4.5), 6.3 (IQR: 5.9–6.6), 12.4 (IQR: 12.0-12.7), and 23.9 (IQR: 23.6–24.2) months respectively post-SARS-CoV-2 infection.

### Associations between socio-demographic, health and educational needs status prior to infection and subsequent PCC trajectories

Female sex and older age at infection were consistently associated with increased risk of several PCC trajectories. For example, females had higher relative risk of chronic PCC (RRR:2.78 95% CI:1.45–5.33), fluctuating PCC (1.58, 95% CI:1.01,2.47), and late onset PCC (1.88, 95% CI:1.31,2.69); but, the risk of females being in the recovered PCC trajectory was the same as in never PCC (reference) trajectory (Table 2). There was little/inconsistent evidence of associations between ethnicity, region, socioeconomic position and PCC trajectories, with confidence intervals generally wide and including the null. In contrast, pre-pandemic physical and mental health were both strongly associated with PCC trajectories such that poor-to-ok physical health prior to infection was associated with increased risk of chronic (2.98, 95% CI:1.72,5.15), fluctuating (2.56, 95% CI:1.63,4.03), and late onset PCC (1.92, 95% CI:1.31–2.81). Poor-to-ok mental health showed even stronger associations. Indicators of prior healthcare use and additional needs at school were also strongly associated with PCC trajectories, particularly the chronic and fluctuating PCC trajectories.

Overall, the strongest and most consistent associations with adverse PCC trajectories (in particular chronic and fluctuating) were female sex, older age, poorer pre-infection mental and physical health, prior healthcare use, and educational needs. Other sociodemographic factors showed more modest and inconsistent associations.

### Associations between nature, number, functional impact and severity of symptoms, mental health and wellbeing over the 2-year period following infection and concurrent PCC trajectories

#### Nature of symptoms

There were associations between the number of times CYP reported each symptom across the two-year period and the concurrent PCC trajectories (*p* < 0.0001, Table S2). Fatigue was a prominent, persistent symptom across all groups, with more CYP with Chronic PCC reporting it at all four timepoints (73.53%) compared to those with other PCC trajectories (4.29–32.48%). Likewise, there was a higher prevalence of persistent shortness of breath (36.76%) and persistent headache (14.71%) amongst CYP with Chronic PCC (persistent shortness of breath ranged from 0.0 to 13.68% and persistent headache from 0.0 to 5.98% in the other four PCC trajectory groups). Although prevalences were low, it is noteworthy that only CYP with Chronic PCC reported persistent chest pain (*n* = 5, 7.35%) as well as brain fog, fever, muscle pain and sore throat (*n* = 1, 1.47% for each symptom).

#### Number of symptoms

Consistently those in the Chronic PCC group reported more symptoms at all time points compared to the other groups (Table 3). For example, the median (IQR) number of reported symptoms 24-months post-infection was 5 (3.5-8) for the Chronic PCC group compared to 0 (0–1) in the Never PCC group, 1 (0–2) in the Recovered PCC group and 2 (1–4) in the Fluctuating PCC and late onset PCC groups. Importantly, nobody in the Chronic PCC group had PCC because of a single symptom persisting across time, that is, they always had multiple symptoms as opposed to just one.

#### Functional impact and severity of symptoms

There were associations between hospital attendance for and doctor consultations about COVID-19 and concurrent PCC trajectories (*p* ≤ 0.02; Fishers Exact Test) (Table 4). For example, more CYP with Chronic PCC reported having attended the hospital for their COVID-19 (11.76%) compared to the other PCC trajectories (0.0-6.84%). There were only few overnight hospitalisations, and this was not associated with PCC trajectories.

There was no association between symptom severity and PCC trajectories in the sub-sample with data at 12-months; but there was a difference at 24-months post-infection. For example, median (IQR) symptom severity 12-months post-infection ranged from 50 in the Never PCC (IQR: 30–60), First PCC after 3-months (IQR: 40–70), and Fluctuating PCC (IQR: 40–65) groups to 65 (IQR: 30–70) in the Recovered PCC group (*p* = 0.4482, Kruskal-Wallis Test). 24-months post-infection, median (IQR) symptom severity ranged from 40 (IQR: 30–60) in the Never PCC group to 60 in the late onset PCC (IQR: 40–70) and Chronic PCC (IQR: 50–80) groups (*p* < 0.001, Kruskal-Wallis Test). Findings were similar for reported functional impact. For example, median (IQR) functional impact 12-months post-infection was 30 (IQR: 10–60) in the Never PCC, 35 (IQR: 10–65) in the Fluctuating PCC, 40 in the Chronic PCC (IQR: 10–70) and Recovered PCC (IQR: 20–60) groups, with those first having PCC after 3-months the most impaired, reporting a median of 45 (IQR:10–65) functional impact (*p =* 0. 5681, Kruskal-Wallis Test). At 24-months, median (IQR) functional impact was 35 (IQR: 10–50) in the Recovered, 40 (IQR: 10–60) in the Never, 50 (IQR: 30–70) in the first PCC after 3-months, and 60 in the Chronic (IQR: 40–80) and Fluctuating (IQR: 20–80) PCC groups (*p* < 0.001, Kruskal-Wallis Test).

#### Mental health and wellbeing

There was an association between frequency of caseness on almost all SDQ scales and concurrent PCC trajectories such that the prevalence of those consistently reaching caseness was highest in CYP with Chronic PCC (Table 5). For example, 41.18% of Chronic, 17.09% of Fluctuating, 8.33% of Recovered, 5.91% of late onset PCC, and 1.63% of Never PCC persistently reached caseness (as per the SDQ total score) over the 2-year follow up period (*p* < 0.0001). With the exception of the SDQ Prosocial scale, similar patterns were observed for the other SDQ subscales with the Chronic PCC group having the highest percentage of CYP continuously reaching caseness. This denotes mental health difficulties continuously impairing this group more. Similar to SDQ caseness patterns, a higher proportion of the Chronic PCC group (14.71%) reported low mental wellbeing (as measured by SWEMWS) consistently throughout the follow-up period compared to the other PCC trajectories where percentages ranged from 0.61% (Never PCC) to 5.98% (Fluctuating PCC) (*p* < 0.0001).

## Discussion

In this study, examining 943 CYP over two years post-infection, we had two important findings. First, we found that baseline factors prior to the COVID-19 pandemic/infection such as female sex, older age, poorer pre-infection mental and physical health, prior healthcare use, and educational needs were strongly and consistently associated with adverse PCC trajectories. For example, compared to those aged 11-to-14-years at infection, those aged 15-to-17-years had a 2.44 higher risk of being in the Chronic PCC group (compared to the Never PCC group). Similarly, the risk of being in the Fluctuating PCC group was 1.57, the Recovered PCC group was 2.08 and the late onset PCC group was 1.50. Other sociodemographic factors, such as ethnicity and region of residence, showed more modest and inconsistent associations. Secondly, the PCC trajectories differed by concurrent number, frequency, functional impact and severity of symptoms and mental health. For example, CYP with Chronic PCC consistently reported a higher median number of symptoms (5 + at each timepoint) compared to the other PCC groups (where the median number of symptoms was always ≤ 3). Likewise, the mental health and wellbeing, of the Chronic PCC group was consistently worse than that of the other groups, with 41.18% of the Chronic PCC group reaching caseness on the SDQ at all time-points, compared to 17.09%-to-1.63% of the other groups.

Our findings contribute to understanding which CYP: recover from PCC, have a fluctuating illness course or experience Chronic PCC. While, to our knowledge, no previous work has examined heterogeneity in PCC trajectories over a two year period in CYP as we have, consistencies with previous literature are found. For example, female sex and older age were highlighted as risk factors for PCC [36–39]. Elsewhere, including in previous CLoCk studies [40], poorer prior physical and mental health were associated with subsequent PCC. CYP with no pre-existing conditions (e.g., ADHD, Anxiety, Heart Disease, Asthma) [41] were less likely to present with Chronic and Fluctuating PCC. Relatedly, CYP seeking treatment from PCC hubs were more likely to report prior health problems [42]. We found that almost one in five of the Chronic PCC group reported learning difficulties, indicating that children with learning difficulties may be particularly vulnerable to PCC becoming chronic. This is consistent with data from Israel suggesting neurodiversity is associated with worse post-infection outcomes, including PCC [43, 44]. Importantly, these findings should be interpreted as associations only. We do not infer causality, as alternative explanations remain plausible, including differences in symptom perception, reporting behaviour, or underlying health vulnerability.

In terms of symptomology concurrent with PCC trajectories, we found that fatigue was a prominent, consistent, persistent symptom across all PCC groups. Importantly, all CYP with Chronic PCC reported feeling fatigued at least once throughout the two-year period, with 73.5% (50/68) feeling fatigued at all time-points. This is consistent with previous literature where fatigue is highlighted as a common symptom in PCC [6–8, 41], persisting over a 12 month period [21] and remaining prevalent even two years post-infection [20]. Similarly, the Chronic PCC group had the highest percentage of CYP with continuous shortness of breath and headache reflecting previous findings [6–8]. The median number of symptoms at each timepoint was consistently higher for the Chronic PCC group (5+) compared to the others, having implications for the definition of PCC, which can be met by the presence of just one impairing symptom. These data suggest those with Chronic PCC often experience multiple symptoms and raises the question of whether definitions need revising to prevent overestimates of PCC prevalence and/or reduce heterogeneity (i.e., in symptoms required to meet the criteria). A higher percentage of CYP in the Chronic PCC group reported attending a hospital for their COVID-19 than other PCC groups, which is consistent with previous literature showing hospital attendance predicts COVID-19 outcomes [45]. The mental health and wellbeing of the Chronic PCC group was consistently worse than that of other PCC groups, aligning with previous studies that report changes in mood [41] and associations between PCC and anxiety disorders [46], as well as a meta-analysis showing those with PCC being twice as likely to have anxiety or depression compared to those without PCC [47]. The findings speak to the value of having a holistic perspective on physical and mental health rather than separate silos given the strong interconnection between symptoms.

### Methodological considerations and limitations

While other studies have recognised the fluctuating nature of SARS-CoV-2 symptoms, to our knowledge, in-depth comparisons of health across distinct PCC trajectories in CYP have not previously been undertaken. Hence, a strength of the present study is that it addresses this research gap, employing a longitudinal approach with a relatively large sample size of 943 PCR-test positive CYP followed over two years. The use of validated, standardised wellbeing measures constitutes another strength. However, limitations are acknowledged. The study period (2021–2023) overlaps with the end of free community testing in England (April 2022), meaning that reinfections may have not been fully captured. This is particularly relevant for the late onset PCC group, which may in some cases reflect subsequent infection which, in turn, has been associated with more symptoms [20]. Without systematic testing, we are unable to distinguish symptoms due to reinfection from ongoing or new symptom development. This may have introduced some misclassification across trajectory groups, potentially attenuating observed differences. The original CLoCk study included PCR-negative CYP at study invitation; however, these were excluded from this manuscript by design. This reflects uncertainty regarding infection status over time, as PCR-negative participants may subsequently have become infected during follow-up. Our focus on PCR-positive CYP ensures confirmed infection at baseline, although we cannot rule out reinfection during follow-up. Accordingly, PCC findings should be interpreted in the context of infections occurring predominantly during periods when wild-type and Alpha (B.1.1.7) variants were circulating. Selection bias may exist in our study. Approximately 4% of the target population of invited test-positives were part of the examined analytical sample, and these CYP may not be representative of the broader population of CYP who PCR-tested positive for COVID-19 in England, between January and March 2021. Selection bias, as well as systematic differences in attrition after enrolment, may affect the generalisability of the findings. Although methods exist to account for such biases, they rely on assumptions that cannot be fully verified. Reassuringly, the analytical sample was broadly demographically similar to the broader cohort of PCR-positive CYP invited to enrol in the CLoCk study. Another relevant limitation is that by relying on four distinct data collection sweeps, we are uncertain of PCC status between these sweeps, leading to potential misclassification of PCC trajectories. Future linkages to electronic health records may help reduce this misclassification. Additionally, future studies could also implement more sophisticated modelling approaches (e.g., latent class trajectory analysis or growth mixture modelling) to further understand the natural course of PCC. However, this was beyond the scope of the present study as there is a need to first understand and describe these CYP and the PCC trajectory patterns on a more simplistic level. Symptoms reported at each timepoint may not be a direct consequence of SARS-CoV-2 and instead might reflect a current cold/illness. Finally, retrospective reporting of health and educational needs prior to infection needs to be considered in light of its potential to introduce recall bias.

## Conclusions

In conclusion, this study was designed to understand differences between the different trajectories of PCC that CYP might take over 2-years post-infection. We found consistent differences between PCC trajectories, particularly in terms of neurodiversity, and number and type of symptoms but not the impact and severity of those symptoms. Understanding the differential trajectories of CYP and the natural course of PCC will help with revised definitions of PCC and treatment planning.

**Table 1:** Socio-demographics, health and educational needs (prior to infection) of Polymerase Chain Reaction (PCR) positive children and young people invited, enrolled and part of the analytical sample (N(%))

	PCR-Positives invited* (*N* = 23,048)	PCR-Positives enrolled 3-months post-infection (*N* = 3,321)	
		Not part of analytical sample (*N* = 2,378)	Analytical sample (*N* = 943)
**Socio-demographics**			
Sex at birth			
Male	10,636 (46.15)	938 (39.44)	298 (31.60)
Female	12,412 (53.85)	1,440 (60.56)	645 (68.40)
Age at infection (Years)			
11–14	10,651 (46.21)	976 (41.04)	401 (42.52)
15–17	12,397 (53.79)	1,402 (58.96)	542 (57.48)
Ethnicity**			
White	-	1,720 (72.33)	698 (74.02)
Asian/Asian British	-	391 (16.44)	148 (15.69)
Black/African/Caribbean/British	-	84 (3.53)	34 (3.61)
Mixed	-	113 (4.75)	42 (4.45)
Other	-	48 (2.02)	16 (1.70)
Prefer not to say	-	22 (0.93)	5 (0.53)
Region of England			
East Midlands	1,815 (7.87)	227 (9.55)	92 (9.76)
East of England	3,392 (14.72)	362 (15.22)	139 (14.74)
London	4,412 (19.14)	408 (17.16)	137 (14.53)
North East England	819 (3.55)	84 (3.53)	31 (3.29)
North West England	3,235 (14.04)	295 (12.41)	111 (11.77)
South East England	3,496 (15.17)	360 (15.14)	169 (17.92)
South West England	1,238 (5.37)	169 (7.11)	88 (9.33)
West Midlands	2,854 (12.38)	292 (12.28)	118 (12.51)
Yorkshire and the Humber	1,787 (7.75)	181 (7.61)	58 (6.15)
Index of Multiple Deprivation (IMD) quintile			
1 (most deprived)	6,732 (29.21)	530 (22.29)	167 (17.71)
2	5,198 (22.55)	488 (20.52)	187 (19.83)
3	4,159 (18.04)	445 (18.71)	177 (18.77)
4	3,679 (15.96)	453 (19.05)	188 (19.94)
5 (least deprived)	3,280 (14.23)	462 (19.43)	224 (23.75)
Pre-pandemic health**			
Pre-pandemic Physical Health**			
Very poor/poor	-	50 (2.10)	19 (2.01)
Ok	-	516 (21.70)	194 (20.57)
Very good/good	-	1,812 (76.20)	730 (77.41)
Pre-pandemic Mental Health**			
Very poor/poor	-	211 (8.87)	87 (9.23)
Ok	-	694 (29.18)	268 (28.42)
Very good/good	-	1,473 (61.94)	588 (62.35)
Pre-pandemic Mental Health Treatment**			
No	-	2,155 (90.62)	853 (90.46)
Yes	-	223 (9.38)	90 (9.54)
Prescription from doctor prior to COVID-19 infection**			
No	-	2,202 (92.60)	871 (92.36)
Yes	-	176 (7.40)	72 (7.64)
Pre-pandemic educational Needs**			
Learning Difficulties**			
No	-	2,167 (91.13)	890 (94.38)
Yes	-	211 (8.87)	53 (5.62)
Education, Health and Care Plan (EHCP)**			
No	-	2,233 (93.90)	907 (96.18)
Yes	-	145 (6.10)	36 (3.82)

*invited 3-months post-infection; **self-reported at enrolment

**Table 2:** Associations* between socio-demographics, health and educational needs (prior to infection) and Post Covid-19 Condition (PCC) trajectories: Relative risk ratios (95% Confidence Intervals)

	Chronic PCC (*N* = 68)	Fluctuating PCC (*N* = 117)	Recovered PCC (*N* = 48)	Late onset PCC (*N* = 220)
Sex at birth (female vs male)	2.78 (1.45,5.33)	1.58 (1.01,2.47)	0.99 (0.54,1.83)	1.88 (1.31,2.69)
Age at infection (15–17 vs. 11–14)	2.44 (1.39,4.26)	1.57 (1.04,2.37)	2.08 (1.10,3.92)	1.50 (1.08,2.07)
Ethnicity (ref: White)**				
Asian/Asian British	0.63 (0.29,1.45)	1.22 (0.70,2.11)	0.97 (0.42,2.26)	1.28 (0.83,1.96)
Black/African/Caribbean/British	0.35 (0.05,2.69)	0.71 (0.21,2.46)	1.08 (0.24,4.82)	1.12 (0.50,2.53)
Mixed/Other	1.34 (0.49,3.65)	1.98 (0.94,4.19)	0.82 (0.19,3.60)	1.42 (0.73,2.77)
Region of England (ref: London)				
East Midlands	0.44 (0.14,1.46)	2.17 (0.84,5.62)	0.33 (0.04,3.07)	0.68 (0.37,1.27)
East of England	0.81 (0.33,2.00)	1.82 (0.73,4.57)	3.16 (0.98,10.16)	0.67 (0.38,1.18)
North East England	0.28 (0.03,2.31)	1.27 (0.31,5.26)	-	0.53 (0.21,1.35)
North West England	0.57 (0.20,1.60)	3.12 (1.29,7.52)	0.57 (0.10,3.20)	0.53 (0.28,0.99)
South East England	0.86 (0.37,2.00)	1.78 (0.73,4.34)	2.97 (0.94,9.34)	0.65 (0.38,1.11)
South West England	1.55 (0.64,3.75)	2.32 (0.88,6.13)	0.77 (0.14,4.40)	0.62 (0.32,1.20)
West Midlands	0.78 (0.30,2.04)	2.49 (1.00,6.19)	2.93 (0.87,9.84)	0.65 (0.36,1.19)
Yorkshire and the Humber	0.34 (0.07,1.62)	2.32 (0.82,6.55)	0.52 (0.06,4.79)	0.60 (0.28,1.25)
Index of Multiple Deprivation (IMD) quintile (ref: quintile 1 (most deprived))				
2	1.19 (0.51,2.76)	0.56 (0.28,1.10)	2.48 (0.66,9.30)	0.54 (0.33,0.90)
3	1.82 (0.79,4.20)	1.39 (0.75,2.57)	2.70 (0.69,10.56)	0.80 (0.48,1.34)
4	1.01 (0.41,2.45)	0.84 (0.44,1.59)	3.91 (1.09,14.12)	0.73 (0.44,1.20)
5 (least deprived)	0.68 (0.28,1.65)	0.43 (0.22,0.84)	2.47 (0.68,8.93)	0.48 (0.30,0.79)
Physical Health (ref: Very good/good)				
Very poor/poor/Ok	2.98 (1.72,5.15)	2.56 (1.63,4.03)	1.18 (0.55,2.54)	1.92 (1.31,2.81)
Mental Health (ref: Very good/good)				
Very poor/poor/Ok	8.85 (4.93,15.90)	3.11 (2.05,4.71)	2.30 (1.25,4.21)	2.41 (1.73,3.37)
Mental Health Treatment	8.46 (4.20,17.04)	4.00 (2.02,790)	9.68 (4.50,20.83)	2.48 (1.31,4.68)
Prescription from doctor prior to COVID-19 infection	6.09 (2.91,12.75)	3.72 (1.86,7.43)	1.02 (0.23,4.51)	2.35 (1.24,4.46)
Learning Difficulties	8.67 (3.83,19.65)	3.81 (1.66,8.73)	1.60 (0.35,7.29)	2.49 (1.15,5.40)
Education, Health and Care Plan (EHCP)	7.32 (2.86,18.75)	3.05 (1.14,8.20)	1.02 (0.13,8.15)	2.05 (0.82,5.11)

*Univariate associations [reference PCC trajectory: Never PCC]; ** analysis excludes 5 children and young people that chose ‘prefer not to say’

**Table 3:** Median (25th, 75th centile) number of symptoms reported at each time point post-infection by Post Covid-19 Condition (PCC) trajectory

months post-infection	Never PCC (*n* = 490)	Chronic PCC (*n* = 68)	Fluctuating PCC (*n* = 117)	Recovered PCC (*n* = 48)	Late onset PCC (*n* = 220)
3 months	0 (0-2)	5 (3-6)	3 (2-5)	3 (2-5)	1.5 (0-3)
6 months	0 (0–1)	5 (3–7)	3 (1–5)	1 (0–3)	2 (1–4)
12 months	0 (0–1)	6 (4-8.5)	3 (1–5)	1 (0-3.5)	3 (2–5)
24 months	0 (0–1)	5 (3.5-8)	2 (1–4)	1 (0–2)	2 (1–4)

**Table 4:** Frequency (N(%)) of children and young people reporting hospital attendance, overnight hospitalisation and seeking a doctor for their COVID-19 over 2-years post-infection, stratified by concurrent 2-year Post Covid-19 Condition (PCC) trajectory

	Never PCC (*N* = 490)	Chronic PCC (*N* = 68)	Fluctuating PCC (*N* = 117)	Recovered PCC (*N* = 48)	Late onset PCC (*N* = 220)	*p*-value
Hospital attendance for COVID-19						<0.001
No	480 (97.96)	60 (88.24)	109 (93.16)	48 (100.00)	215 (97.73)	
Yes	10 (2.04)	8 (11.76)	8 (6.84)	0 (0.00)	5 (2.27)	
Overnight hospitalisation due to COVID-19						0.46
No	480 (97.96)	65 (95.59)	113 (96.58)	48 (100.00)	216 (98.18)	
Yes	10 (2.04)	3 (4.41)	4 (3.42)	0 (0.00)	4 (1.82)	
Doctor consultation about COVID-19						
No	423 (86.33)	49 (72.06)	98 (83.76)	37 (77.08)	178 (80.91)	0.02
Yes	67 (13.67)	19 (27.94)	19 (16.24)	11 (22.92)	42 (19.09)	

* p-values from Fishers Exact test

**Table 5:** Frequency (N (%)) of reaching caseness on the Strengths and Difficulties Questionnaire (SDQ; and associated subscales) and reporting ‘low mental wellbeing’ on the Short Warwick-Edinburgh Mental Wellbeing Scale (SWEMWS) over 2-years post-infection, by concurrent Post Covid-19 Condition (PCC) trajectory

	Never PCC (*N* = 490)	Chronic PCC (*N* = 68)	Fluctuating PCC (*N* = 117)	Recovered PCC (*N* = 48)	Late onset PCC (*N* = 220)	*p*-value*
**Frequency reaching caseness on:**						
SDQ Total						
0	400 (81.63)	14 (20.59)	53 (45.30)	27 (56.25)	126 (57.27)	< 0.0001
1–3	82 (16.73)	26 (38.24)	44 (37.61)	17 (35.42)	81 (36.82)	
4	8 (1.63)	28 (41.18)	20 (17.09)	4 (8.33)	13 (5.91)	
SDQ Impact*						
0	387 (81.47)	2 (3.28)	43 (38.05)	24 (54.55)	105 (49.30)	< 0.0001
1–3	82 (17.26)	36 (59.02)	57 (50.44)	20 (45.45)	94 (44.13)	
4	6 (1.26)	23 (37.70)	13 (11.50)	0 (0.00)	14 (6.57)	
SDQ Emotional						
0	352 (71.84)	6 (8.82)	36 (30.77)	18 (37.50)	86 (39.09)	< 0.0001
1–3	121 (24.69)	32 (47.06)	55 (47.01)	26 (54.17)	104 (47.27)	
4	17 (3.47)	30 (44.12)	26 (22.22)	4 (8.33)	30 (13.64)	
SDQ Conduct						
0	445 (90.82)	46 (67.65)	85 (72.65)	40 (83.33)	187 (85.00)	< 0.0001
1–3	45 (9.18)	19 (27.94)	31 (26.50)	7 (14.58)	31 (14.09)	
4	0 (0.00)	3 (4.41)	1 (0.85)	1 (2.08)	2 (0.91)	
SDQ Prosocial						
0	362 (73.88)	44 (64.71)	81 (69.23)	35 (72.92)	156 (70.91)	0.0591^a^
1–3	117 (23.88)	22 (32.35)	30 (25.64)	10 (20.83)	58 (26.36)	
4	11 (2.24)	2 (2.94)	6 (5.13)	3 (6.25)	6 (2.73)	
SDQ Hyperactivity						
0	397 (81.02)	18 (26.47)	65 (55.56)	30 (62.50)	123 (55.91)	< 0.0001
1–3	85 (17.35)	30 (44.12)	41 (35.04)	13 (27.08)	85 (38.64)	
4	8 (1.63)	20 (29.41)	11 (9.40)	5 (10.42)	12 (5.45)	
SDQ Peer						
0	328 (66.94)	21 (30.88)	48 (41.03)	23 (47.92)	114 (51.82)	< 0.0001
1–3	144 (29.39)	27 (39.71)	50 (42.74)	24 (50.00)	89 (40.45)	
4	18 (3.67)	20 (29.41)	19 (16.24)	1 (2.08)	17 (7.73)	
**Frequency reporting low wellbeing on:**						
SWEMWS						
0	422 (86.12)	24 (35.29)	72 (61.54)	31 (64.58)	133 (60.45)	< 0.0001
1–3	65 (13.27)	34 (50.00)	38 (32.48)	15 (31.25)	81 (36.82)	
4	3 (0.61)	10 (14.71)	7 (5.98)	2 (4.17)	6 (2.73)	

Note. SDQ caseness was determined using established cut offs for self-reported SDQ scores [48]. Those scoring ≥ 18 (total difficulties); ≥2 (impact); ≥6 (emotional symptoms), ≥ 5 (conduct problems), ≤ 5 (prosocial skills); ≥7 (hyperactivity) and ≥ 4 (peer difficulties) are classified as having scored ‘high/very high’ problem/impact. *O*n SWEMWS, ‘low’ mental wellbeing was regarded as a score ≤ 17, which falls between the expected range of UK population samples (7.0-19.5) [49]

N for SDQ Impact varies due to missing data; N in each group is: 475, 61, 113, 44 and 213 respectively

*p-values are for Chi-Square tests/Fishers Exact (denoted by ^a^) after correction for multiple testing via Bonferroni adjustment (number of tests=8; i.e., p<0.05 refers to p<0.00625 and p<0.01 refers to p<0.00125)

## Supplementary Information

Below is the link to the electronic supplementary material.

Supplementary Material 1: Description of data: This document includes Table S1 which provides further information on the variables used and their handling in the present study and Table S2 which provides information on the frequency (N(%)) of CYP reporting each symptom over 2-years post-infection, stratified by concurrent 2-year PCC trajectory.

## Data Availability

CLoCk data is publicly available and can be accessed on the UK Data Service: https://beta.ukdataservice.ac.uk/datacatalogue/studies/study?id=9203.

## References

[1] Ng CSM, Ng SSL. Impact of the COVID-19 pandemic on children's mental health: A systematic review. Front Psychiatry. 2022;13. 10.3389/fpsyt.2022.975936 PMC9622998 36329921

[2] A clinical case definition for post COVID-19 condition in children and adolescents by expert consensus, 16. February 2023. https://www.who.int/publications/i/item/WHO-2019-nCoV-Post-COVID-19-condition-CA-Clinical-case-definition-2023-1.

[3] Stephenson T, Allin B, Nugawela MD, Rojas N, Dalrymple E, Pinto Pereira S, Soni M, Knight M, Cheung EY, Heyman I, et al. Long COVID (post-COVID-19 condition) in children: a modified Delphi process. Arch Dis Child. 2022;107(7):674–80. 10.1136/archdischild-2021-323624 PMC8983414 35365499

[4] Pan D, Pareek M. Toward a universal definition of post–COVID-19 condition—how do we proceed? JAMA Netw Open. 2023;6(4). 10.1001/jamanetworkopen.2023.5779 37017975

[5] Chaichana U, Man KKC, Chen A, Wong ICK, George J, Wilson P, Wei L. Definition of Post-COVID-19 Condition Among Published Research Studies. JAMA Netw Open. 2023;6(4):e235856. 10.1001/jamanetworkopen.2023.5856 PMC10077105 37017970

[6] Behnood S, Newlands F, O’Mahoney L, Haghighat Ghahfarokhi M, Muhid MZ, Dudley J, Stephenson T, Ladhani SN, Bennett S, Viner RM, et al. Persistent symptoms are associated with long term effects of COVID-19 among children and young people: Results from a systematic review and meta-analysis of controlled studies. PLoS ONE. 2023;18(12):e0293600. 10.1371/journal.pone.0293600 PMC10754445 38153928

[7] Pellegrino R, Chiappini E, Licari A, Galli L, Marseglia GL. Prevalence and clinical presentation of long COVID in children: a systematic review. Eur J Pediatr. 2022;181(12):3995–4009. 10.1007/s00431-022-04600-x PMC9476461 36107254

[8] Zheng YB, Zeng N, Yuan K, Tian SS, Yang YB, Gao N, Chen X, Zhang AY, Kondratiuk AL, Shi PP, et al. Prevalence and risk factor for long COVID in children and adolescents: A meta-analysis and systematic review. J Infect Public Health. 2023;16(5):660–72. 10.1016/j.jiph.2023.03.005 PMC9990879 36931142

[9] Menni C, Valdes AM, Freidin MB, Sudre CH, Nguyen LH, Drew DA, Ganesh S, Varsavsky T, Cardoso MJ, El-Sayed Moustafa JS, et al. Real-time tracking of self-reported symptoms to predict potential COVID-19. Nat Med. 2020;26(7):1037–40. 10.1038/s41591-020-0916-2 PMC7751267 32393804

[10] Espin E, Yang C, Shannon CP, Assadian S, He D, Tebbutt SJ. Cellular and molecular biomarkers of long COVID: a scoping review. EBioMedicine. 2023;91:104552. 10.1016/j.ebiom.2023.104552 PMC10082390 37037165

[11] Shachar-Lavie I, Shorer M, Segal H, Fennig S, Ashkenazi-Hoffnung L. Mental health among children with long COVID during the COVID-19 pandemic. Eur J Pediatr. 2023;182(4):1793–801. 10.1007/s00431-023-04854-z PMC9925927 36786887

[12] Panagi L, White SR, Pinto Pereira SM, Nugawela MD, Heyman I, Sharma K, Stephenson T, Chalder T, Rojas NK, Dalrymple E, et al. Mental health in the COVID-19 pandemic: A longitudinal analysis of the CLoCk cohort study. PLoS Med. 2024;21(1):e1004315. 10.1371/journal.pmed.1004315 PMC10807843 38266043

[13] Spurrier GF, Shulman K, Dibich S, Benoit L, Duckworth K, Martin A. Physical symptoms as psychiatric manifestations in medical spaces: A qualitative study. Front Psychiatry. 2022;13:1074424. 10.3389/fpsyt.2022.1074424 PMC9845882 36683974

[14] Richardson LP, Lozano P, Russo J, McCauley E, Bush T, Katon W. Asthma Symptom Burden: Relationship to Asthma Severity and Anxiety and Depression Symptoms. Pediatrics. 2006;118(3):1042–51. 10.1542/peds.2006-0249 16950996

[15] Chavira DA, Garland AF, Daley S, Hough RL. The impact of medical comorbidity on mental health and functional health outcomes among children with anxiety disorders. J Dev Behav Pediatr. 2008;29(5). 10.1097/DBP.0b013e3181836a5b PMC2626436 18714205

[16] Butwicka A, Frisén L, Almqvist C, Zethelius B, Lichtenstein P. Risks of Psychiatric Disorders and Suicide Attempts in Children and Adolescents With Type 1 Diabetes: A Population-Based Cohort Study. Diabetes Care. 2015;38(3):453. 10.2337/dc14-0262 PMC4338504 25650362

[17] Adams JS, Chien AT, Wisk LE. Mental illness among youth with chronic physical conditions. Pediatrics. 2019;144(1). 10.1542/peds.2018-1819 PMC12930273 31201229

[18] Long-term conditions and mental. health: the cost of co-morbidities. https://www.kingsfund.org.uk/sites/default/files/field/field_publication_file/long-term-conditions-mental-health-cost-comorbidities-naylor-feb12.pdf.

[19] Nugawela MD, Pinto Pereira SM, Rojas NK, McOwat K, Simmons R, Dalrymple E, et al. Data resource profile: the Children and Young People with Long COVID (CLoCk) Study. Int J Epidemiol. 2024;53(1). 10.1093/ije/dyad158 PMC10859131 37990948

[20] Stephenson T, Pinto Pereira SM, Nugawela MD, Dalrymple E, Harnden A, Whittaker E, Heyman I, Ford T, Segal T, Chalder T, et al. A 24-month National Cohort Study examining long-term effects of COVID-19 in children and young people. Commun Med (Lond). 2024;4(1):255. 10.1038/s43856-024-00657-x PMC11618575 39633013

[21] Pinto Pereira SM, Shafran R, Nugawela MD, Panagi L, Hargreaves D, Ladhani SN, Bennett SD, Chalder T, Dalrymple E, Ford T, et al. Natural course of health and well-being in non-hospitalised children and young people after testing for SARS-CoV-2: a prospective follow-up study over 12 months. Lancet Reg Health Eur. 2023;25:100554. 10.1016/j.lanepe.2022.100554 PMC9719829 36504922

[22] Stephenson T, Shafran R, De Stavola B, Rojas N, Aiano F, Amin-Chowdhury Z, McOwat K, Simmons R, Zavala M, Consortium C, et al. Long COVID and the mental and physical health of children and young people: national matched cohort study protocol (the CLoCk study). BMJ Open. 2021;11(8):e052838. 10.1136/bmjopen-2021-052838 PMC8392739 34446502

[23] English indices of deprivation. 2015. https://www.gov.uk/government/statistics/english-indices-of-deprivation-2015.

[24] Sigfrid L, Maskell K, Bannister PG, Ismail SA, Collinson S, Regmi S, Blackmore C, Harriss E, Longuere KS, Gobat N, et al. Addressing challenges for clinical research responses to emerging epidemics and pandemics: a scoping review. BMC Med. 2020;18(1):190. 10.1186/s12916-020-01624-8 PMC7315698 32586391

[25] Mental Health of Children and Young People in England. 2020: Wave 1 follow up to the 2017 survey. https://digital.nhs.uk/data-and-information/publications/statistical/mental-health-of-children-and-young-people-in-england/2020-wave-1-follow-up.

[26] Children with special educational needs and disabilities (SEND). https://www.gov.uk/children-with-special-educational-needs/extra-SEN-help#:~:text=An%20education%2C%20health%20and%20care,support%20to%20meet%20those%20needs.

[27] Goodman R. Psychometric properties of the strengths and difficulties questionnaire. J Am Acad Child Adolesc Psychiatry. 2001;40(11):1337–45. 10.1097/00004583-200111000-00015 11699809

[28] Short Warwick-Edinburgh Mental Wellbeing Scale (SWEMWS). https://www.corc.uk.net/outcome-experience-measures/short-warwick-edinburgh-mental-wellbeing-scale-swemws/#:~:text=The%20SWEMWBS%20is%20a%20short,aim%20to%20improve%20mental%20wellbeing.

[29] Ringdal R, Bradley Eilertsen ME, Bjørnsen HN, Espnes GA, Moksnes UK. Validation of two versions of the Warwick-Edinburgh Mental Well-Being Scale among Norwegian adolescents. Scand J Public Health. 2018;46(7):718–25. 10.1177/1403494817735391 29017402

[30] McKay MT, Andretta JR. Evidence for the Psychometric Validity, Internal Consistency and Measurement Invariance of Warwick Edinburgh Mental Well-being Scale Scores in Scottish and Irish Adolescents. Psychiatry Res. 2017;255:382–6. 10.1016/j.psychres.2017.06.071 28666244

[31] Ng Fat L, Scholes S, Boniface S, Mindell J, Stewart-Brown S. Evaluating and establishing national norms for mental wellbeing using the short Warwick-Edinburgh Mental Well-being Scale (SWEMWBS): findings from the Health Survey for England. Qual Life Res. 2017;26(5):1129–44. 10.1007/s11136-016-1454-8 PMC5376387 27853963

[32] Koushede V, Lasgaard M, Hinrichsen C, Meilstrup C, Nielsen L, Rayce SB, Torres-Sahli M, Gudmundsdottir DG, Stewart-Brown S, Santini ZI. Measuring mental well-being in Denmark: Validation of the original and short version of the Warwick-Edinburgh mental well-being scale (WEMWBS and SWEMWBS) and cross-cultural comparison across four European settings. Psychiatry Res. 2019;271:502–9. 10.1016/j.psychres.2018.12.003 30551082

[33] Wille N, Badia X, Bonsel G, Burström K, Cavrini G, Devlin N, Egmar AC, Greiner W, Gusi N, Herdman M, et al. Development of the EQ-5D-Y: a child-friendly version of the EQ-5D. Qual Life Res. 2010;19(6):875–86. 10.1007/s11136-010-9648-y PMC2892611 20405245

[34] Ravens-Sieberer U, Wille N, Badia X, Bonsel G, Burstrom K, Cavrini G, Devlin N, Egmar AC, Gusi N, Herdman M, et al. Feasibility, reliability, and validity of the EQ-5D-Y: results from a multinational study. Qual Life Res. 2010;19(6):887–97. 10.1007/s11136-010-9649-x PMC2892614 20401552

[35] Stata Statistical Software. Release 17.

[36] Haddad A, Janda A, Renk H, Stich M, Frieh P, Kaier K, Lohrmann F, Nieters A, Willems A, Huzly D, et al. Long COVID symptoms in exposed and infected children, adolescents and their parents one year after SARS-CoV-2 infection: A prospective observational cohort study. EBioMedicine. 2022;84:104245. 10.1016/j.ebiom.2022.104245 PMC9495281 36155957

[37] Borch L, Holm M, Knudsen M, Ellermann-Eriksen S, Hagstroem S. Long COVID symptoms and duration in SARS-CoV-2 positive children - a nationwide cohort study. Eur J Pediatr. 2022;181(4):1597–607. 10.1007/s00431-021-04345-z PMC8742700 35000003

[38] Molteni E, Sudre CH, Canas LS, Bhopal SS, Hughes RC, Antonelli M, Murray B, Klaser K, Kerfoot E, Chen L, et al. Illness duration and symptom profile in symptomatic UK school-aged children tested for SARS-CoV-2. Lancet Child Adolesc Health. 2021;5(10):708–18. 10.1016/S2352-4642(21)00198-X PMC8443448 34358472

[39] Stephenson T, Pinto Pereira SM, Shafran R, de Stavola BL, Rojas N, McOwat K, Simmons R, Zavala M, O’Mahoney L, Chalder T, et al. Physical and mental health 3 months after SARS-CoV-2 infection (long COVID) among adolescents in England (CLoCk): a national matched cohort study. Lancet Child Adolesc Health. 2022;6(4):230–9. 10.1016/S2352-4642(22)00022-0 PMC8820961 35143770

[40] Nugawela MD, Stephenson T, Shafran R, De Stavola BL, Ladhani SN, Simmons R, McOwat K, Rojas N, Dalrymple E, Cheung EY, et al. Predictive model for long COVID in children 3 months after a SARS-CoV-2 PCR test. BMC Med. 2022;20(1):465. 10.1186/s12916-022-02664-y PMC9708506 36447237

[41] Buonsenso D, Espuny Pujol F, Munblit D, Pata D, McFarland S, Simpson FK. Clinical characteristics, activity levels and mental health problems in children with long coronavirus disease: a survey of 510 children. Future Microbiol. 2022;17(8):577–88. 10.2217/fmb-2021-0285 PMC9248023 35360923

[42] Newlands F, Goddings AL, Juste M, Boyd H, Nugawela MD, Pinto Pereira SM, Whelan E, Whittaker E, Stephenson T, Heyman I, et al. Children and young people with long COVID—comparing those seen in post-COVID services with a non-hospitalised national cohort: a descriptive study. Children (Basel). 2023;10(11) 10.3390/children10111750 PMC10670307 38002841

[43] Merzon E, Weiss MD, Cortese S, Rotem A, Schneider T, Craig SG, Vinker S, Golan Cohen A, Green I, Ashkenazi S, et al. The Association between ADHD and the Severity of COVID-19 Infection. J Atten Disord. 2022;26(4):491–501. 10.1177/10870547211003659 33797281

[44] Merzon E, Weiss M, Krone B, Cohen S, Ilani G, Vinker S, Cohen-Golan A, Green I, Israel A, Schneider T, et al. Clinical and socio-demographic variables associated with the diagnosis of long COVID syndrome in youth: a population-based study. Int J Environ Res Public Health. 2022;19(10). 10.3390/ijerph19105993 PMC9141083 35627530

[45] Maestre-Muniz MM, Arias A, Mata-Vazquez E, Martin-Toledano M, Lopez-Larramona G, Ruiz-Chicote AM, Nieto-Sandoval B, Lucendo AJ. Long-term outcomes of patients with coronavirus disease 2019 at one year after hospital discharge. J Clin Med. 2021;10(13). 10.3390/jcm10132945 PMC8269002 34209085

[46] Kostev K, Smith L, Koyanagi A, Konrad M, Jacob L. Post-COVID-19 conditions in children and adolescents diagnosed with COVID-19. Pediatr Res. 2024;95(1):182–7. 10.1038/s41390-022-02111-x PMC9107066 35568733

[47] Mat Hassan N, Salim HS, Amaran S, Yunus NI, Yusof NA, Daud N, Fry D. Prevalence of mental health problems among children with long COVID: A systematic review and meta-analysis. PLoS ONE. 2023;18(5):e0282538. 10.1371/journal.pone.0282538 PMC10191312 37195978

[48] Scoring. the SDQ. https://www.sdqinfo.org/py/sdqinfo/c0.py.

[49] Collect score. analyse and interpret WEMWBS. https://warwick.ac.uk/fac/sci/med/research/platform/wemwbs/using/howto/.

[50] Research involving children - health research authority. https://www.hra.nhs.uk/planning-and-improving-research/policies-standards-legislation/research-involving-children/.

