# Supplementary material for: Symptom variation up to two years post-SARS-CoV-2 infection in Children and Young People: results from the Children and young people with Long Covid (CLoCk) study
Source: BMC Infect Dis. 2026 Jul 11;26:1776. doi: 10.1186/s12879-026-13951-x (PMC13640280; doi:10.1186/s12879-026-13951-x)
Supplement: Supplementary file 1 [file 12879_2026_13951_MOESM1_ESM.docx]

Supplementary Materials

**Table S1.** Further information on the variables used and their handling in the present study

| Variables | Details | Collection point |
| --- | --- | --- |
| Age at infection | Obtained from UKHSA during study design; dichotomised into 11-14 and 15-17 on the basis of key educational stages. | Study design |
| Sex at birth | Obtained from UKHSA during study design (Male/Female). | Study design |
| Region | Obtained from UKHSA during study design (East Midlands; East of England; London; North East England; North West England; South East England; South West England; West Midlands; Yorkshire and the Humber). | Study design |
| IMD | Derived using CYP’s lower super output area (i.e., small local area level-based geographic hierarchy) where higher values were indicative of lower deprivation [1]. | Study design |
| Ethnicity | Self-reported at enrolment grouped as: Asian or Asian British; Black, African, or Caribbean; Mixed; White; Other; Prefer not to say. | 3-months post infection |
| Prior physical health | CYP were asked about their previous (i.e., before their COVID-19 test) physical health on a five-point scale (Very poor, poor, okay, good, very good); re-grouped into three categories: Very poor/poor, Okay, Very good/good. | 3-months post infection |
| Prior mental health | CYP were asked about their previous (i.e., before their COVID-19 test) mental health on a five-point scale (Very poor, poor, okay, good, very good); re-grouped into three categories: Very poor/poor, Okay, Very good/good. | 3-months post infection |
| Mental health treatment | At enrolment, CYP were asked whether they had previously undergone treatment such as “talking therapies” for their mental health (Yes/No) | 3-months post infection |
| Medication from doctor | At enrolment, CYP were asked whether they were taking any medication (e.g., to help manage their concentration) prescribed by their doctor prior to their COVID-19 test (Yes/No) | 3-months post infection |
| Learning disability | At enrolment, CYP were asked whether they had a learning disability (Yes/No) prior to the COVID-19 pandemic. This was included due to its association with poorer outcomes following SARS-CoV-2 infection and subsequent PCC [2, 3]. | 3-months post infection |
| EHCP | At enrolment, CYP were asked whether they had an Education Health and Care Plan in place before the pandemic (Yes/No). Presence of lack thereof an EHCP was used as proxy for neurodiversity (though it should be noted not all neurodiverse CYP have an EHCP). This was included due to its association with poorer outcomes following SARS-CoV-2 infection and subsequent PCC [2, 3] | 3-months post infection |
| Current symptoms | CYP were asked to select which of a list of 21 symptoms they were experiencing. These were mostly assessed as present/absent and included the following:   1. Fever 2. Chills 3. Persistent cough 4. Tiredness 5. Shortness of Breath 6. Loss of smell and taste 7. Hoarse voice 8. Chest pain 9. Abdominal pain 10. Diarrhoea 11. Headaches 12. Brain fog 13. Eye soreness/discomfort 14. Skipping meals 15. Dizziness or light-headedness 16. Sore throat 17. Muscle pain 18. Earache or ringing in your ears 19. Raised red itchy welts on skin or sudden swelling of face or lips 20. Red/purple sores or blisters on feet/toes 21. Other   Symptom presence throughout the follow up period was considered, with the number of times (0, 1-3, or 4) CYP reported each symptom over the two-year period examined. Sleeping problems were introduced as an additional symptom at 12- and 24-months post-infection (and included to determine whether CYP met the PCC definition or not, as appropriate). | 3-, 6-, 12-, 24-months post infection |
| Quality of life/functioning (EQ-5D-Y) | This scale measures problems (No/Some/A lot of problems) with (a) mobility, (b) self-care, (c) doing usual activities, (d) having pain or discomfort, and (e) feeling worried, sad or unhappy [4]. Used to determine whether CYP met the Delphi definition of PCC (see below ‘PCC trajectories’ for further detail). | 3-, 6-, 12-, 24-months post infection |
| Strengths and Difficulties Questionnaire (SDQ) | Five subscales (25 items) measure: (i) emotional symptoms, (ii) conduct problems, (iii) hyperactivity, (iv) peer relationships, and (v) prosocial skills. Each item was scored from 0 to 2, meaning total scores per subscale ranged from 0-10. A total difficulties score (ranging from 0-40) was derived as the sum of all but the prosocial subscale. The impact of difficulties on CYP is specified by the impact subscale, with scores ranging from 0-10 (higher scores denoting a higher impact).  SDQ caseness was determined using established cut offs for self-reported SDQ scores [5]. Those scoring ≥18 (total difficulties); ≥6 (emotional symptoms), ≥5 (conduct problems), ≥7 (hyperactivity); ≥4 (peer difficulties); ≤5 (prosocial skills), ≥2 (impact) are said to have scored ‘high (or low)/very high (or very low)’. SDQ scores (total and the subscales) throughout the follow up period were considered, with the number of times (0, 1-3, or 4) CYP reached caseness over the two-year period examined. | 3-, 6-, 12-, 24-months post infection |
| Short Warwick Edinburgh Mental Wellbeing Scale (SWEMWS) | Consists of seven items with a five-point scale (None of the time; Rarely; Some of the time; Often; All of the time) scored 1-5. Scores are summed to provide a mental wellbeing score ranging from 7-35. Here, a cut off, derived as one standard deviation below the mean (as recommended; [6]), was used to denote ‘low’ mental wellbeing. These cut off points would be 17.5, 17.4, 17.0, 17.1 for the 3-, 6-, 12-, and 24-month timepoints, respectively. For consistency, ‘low’ mental wellbeing was regarded as a score ≤17 on the SWEMWS, which falls between the expected range of UK population samples (7.0-19.5; [6]). SWEMWS scores throughout the follow up period were considered, with the number of times (0, 1-3, or 4) CYP reported ‘low’ mental wellbeing over the two-year period examined. | 3-, 6-, 12-, 24-months post infection |
| Contacted doctor | CYP were asked if they had ever contacted their doctor due to their COVID-19 (Yes/No). A variable indicating whether they had ever contacted their doctor over the two-year period was derived by combining their answers at 3, 6, 12, and 24 months. | 3-, 6-, 12-, 24-months post infection |
| Attended hospital | CYP were asked if they had ever attended the hospital due to their COVID-19 (Yes/No). A variable indicating whether they had ever attended hospital for their COVID-19 over the two-year period was derived by combining their answers at 3, 6, 12, and 24 months. | 3-, 6-, 12-, 24-months post infection |
| Overnight hospital stay | CYP were asked if they had ever stayed at the hospital overnight due to their COVID-19 (Yes/No). A variable indicating whether they had ever stayed overnight at a hospital for their COVID-19 over the two-year period was derived by combining their answers at 3, 6, 12, and 24 months. | 3-, 6-, 12-, 24-months post infection |
| Symptom severity | CYP were asked “How severe would you rate your symptoms? 0 (not at all) to 100 (extremely)”. There was missing data for this variable with 750/943 and 592/943 CYP not responding at 12 and 24 months, respectively. | 12- and 24-months post infection |
| Functional impact | CYP were asked “How much do your symptoms affect your functioning? 0 (not at all) to 100 (extremely)”. There was missing data for this variable with 751/943 and 594/943 CYP not responding at 12 and 24 months, respectively. | 12- and 24-months post infection |
| PCC trajectories | CYP were assessed in terms of meeting/not meeting the Delphi definition of PCC [7] at each time point as follows:  At least one symptom of the symptoms listed above (under ‘current symptoms’) and at least some or a lot of problems with mobility, self-care, doing usual activities, having pain or discomfort, or feeling very worried, sad or unhappy.  Once PCC at each time-point was determined, we classified CYP into one of five PCC trajectories:   (i) Chronic PCC (i.e., CYP persistently meeting PCC definition at 3-, 6-, 12- and 24-months post-infection) (ii) Fluctuating PCC (i.e., met PCC definition at 3-months with subsequent non-persistent relapses) (iii) Recovered from PCC (i.e., only meeting PCC definition 3-months post-infection) (iv) First had PCC more than 3-months post-infection (i.e., CYP first meeting PCC definition at any timepoint after 3 months either just once, twice or persistently after 3 months) (v) Never had PCC | Derived from data collected at 3-, 6-, 12-, 24-months post infection |

**Table S2.** Frequency (N(%)) of CYP reporting each symptom over 2-years post-infection, stratified by concurrent 2-year PCC trajectory

|  | Never PCC  (N=490) | Chronic PCC  (N=68) | Fluctuating PCC  (N=117) | Recovered PCC  (N=48) | Late onset PCC  (N=220) | p-value* |
| --- | --- | --- | --- | --- | --- | --- |
|  |  |  |  |  |  |  |
| Frequency of CYP reporting: |  |  |  |  |  |  |
|  |  |  |  |  |  |  |
| Fever |  |  |  |  |  |  |
| 0 | 475 (96.94) | 53 (77.94) | 101 (86.32) | 44 (91.67) | 201 (91.36) | <0.0001^a^ |
| 1-3 | 15 (3.06) | 14 (20.59) | 16 (13.67) | 4 (8.33) | 19 (8.64) |  |
| 4 | 0 (0.00) | 1 (1.47) | 0 (0.00) | 0 (0.00) | 0 (0.00) |  |
|  |  |  |  |  |  |  |
| Chills |  |  |  |  |  |  |
| 0 | 434 (88.57) | 25 (36.76) | 75 (64.10) | 36 (75.00) | 139 (63.18) | <0.0001 |
| 1-3 | 56 (11.43) | 39 (57.35) | 38 (32.48) | 11 (22.92) | 75 (34.09) |  |
| 4 | 0 (0.00) | 4 (5.88) | 4 (3.42) | 1 (2.08) | 6 (2.73) |  |
|  |  |  |  |  |  |  |
| Tiredness |  |  |  |  |  |  |
| 0 | 251 (51.22) | 0 (0.00) | 5 (4.27) | 8 (16.67) | 24 (10.91) | <0.0001 |
| 1-3 | 218 (44.49) | 18 (26.47) | 74 (63.25) | 33 (68.74) | 141 (64.09) |  |
| 4 | 21 (4.29) | 50 (73.53) | 38 (32.48) | 7 (14.58) | 55 (25.00) |  |
|  |  |  |  |  |  |  |
| Shortness of breath |  |  |  |  |  |  |
| 0 | 390 (79.59) | 7 (10.29) | 32 (27.35) | 23 (47.92) | 81 (36.82) | <0.0001 |
| 1-3 | 96 (19.59) | 36 (52.94) | 69 (58.98) | 25 (52.09) | 124 (56.36) |  |
| 4 | 4 (0.82) | 25 (36.76) | 16 (13.68) | 0 (0.00) | 15 (6.82) |  |
|  |  |  |  |  |  |  |
| Loss of smell/taste |  |  |  |  |  |  |
| 0 | 427 (87.14) | 47 (69.12) | 79 (67.52) | 31 (64.58) | 171 (77.73) | <0.0001 |
| 1-3 | 60 (12.24) | 17 (25.00) | 37 (31.63) | 15 (31.25) | 49 (22.27) |  |
| 4 | 3 (0.61) | 4 (5.88) | 1 (0.85) | 2 (4.17) | 0 (0.00) |  |
|  |  |  |  |  |  |  |
| Chest pain |  |  |  |  |  |  |
| 0 | 466 (95.10) | 23 (33.82) | 74 (63.25) | 36 (75.00) | 172 (78.18) | <0.0001 |
| 1-3 | 24 (4.90) | 40 (58.82) | 43 (36.76) | 12 (25.00) | 48 (21.82) |  |
| 4 | 0 (0.00) | 5 (7.35) | 0 (0.00) | 0 (0.00) | 0 (0.00) |  |
|  |  |  |  |  |  |  |
| Headache |  |  |  |  |  |  |
| 0 | 368 (75.10) | 11 (16.18) | 33 (28.21) | 22 (45.83) | 90 (40.91) | <0.0001 |
| 1-3 | 120 (24.49) | 47 (69.12) | 77 (65.81) | 26 (54.17) | 122 (55.45) |  |
| 4 | 2 (0.41) | 10 (14.71) | 7 (5.98) | 0 (0.00) | 8 (3.64) |  |
|  |  |  |  |  |  |  |
| Brain fog |  |  |  |  |  |  |
| 0 | 466 (95.10) | 28 (41.18) | 86 (73.50) | 40 (83.33) | 171 (77.73) | <0.0001^a^ |
| 1-3 | 24 (4.90) | 39 (57.35) | 31 (26.49) | 8 (16.66) | 49 (22.27) |  |
| 4 | 0 (0.00) | 1 (1.47) | 0 (0.00) | 0 (0.00) | 0 (0.00) |  |
|  |  |  |  |  |  |  |
| Eye soreness |  |  |  |  |  |  |
| 0 | 463 (94.49) | 32 (47.06) | 74 (63.25) | 36 (75.00) | 171 (77.73) | <0.0001 |
| 1-3 | 27 (5.51) | 36 (52.94) | 42 (35.90) | 12 (25.00) | 48 (21.82) |  |
| 4 | 0 (0.00) | 0 (0.00) | 1 (0.85) | 0 (0.00) | 1 (0.45) |  |
|  |  |  |  |  |  |  |
| Skipping meals |  |  |  |  |  |  |
| 0 | 438 (89.39) | 25 (36.76) | 71 (60.68) | 35 (72.92) | 136 (61.82) | <0.0001 |
| 1-3 | 52 (10.61) | 39 (57.35) | 44 (37.61) | 13 (27.08) | 83 (37.73) |  |
| 4 | 0 (0.00) | 4 (5.88) | 2 (1.71) | 0 (0.00) | 1 (0.45) |  |
|  |  |  |  |  |  |  |
| Dizziness |  |  |  |  |  |  |
| 0 | 419 (85.51) | 17 (25.00) | 53 (45.30) | 29 (60.42) | 126 (57.27) | <0.0001 |
| 1-3 | 71 (14.49) | 45 (66.18) | 63 (53.85) | 19 (39.58) | 90 (40.91) |  |
| 4 | 0 (0.00) | 6 (8.82) | 1 (0.85) | 0 (0.00) | 4 (1.82) |  |
|  |  |  |  |  |  |  |
| Sore throat |  |  |  |  |  |  |
| 0 | 419 (85.51) | 34 (50.00) | 70 (59.83) | 31 (64.58) | 144 (65.45) | <0.0001^a^ |
| 1-3 | 71 (14.49) | 33 (48.53) | 47 (40.18) | 17 (35.41) | 76 (34.55) |  |
| 4 | 0 (0.00) | 1 (1.47) | 0 (0.00) | 0 (0.00) | 0 (0.00) |  |
|  |  |  |  |  |  |  |
| Muscle pain |  |  |  |  |  |  |
| 0 | 480 (97.96) | 28 (41.18) | 82 (70.09) | 43 (89.58) | 177 (80.45) | <0.0001^a^ |
| 1-3 | 10 (2.04) | 39 (57.35) | 35 (29.91) | 5 (10.41) | 43 (19.55) |  |
| 4 | 0 (0.00) | 1 (1.47) | 0 (0.00) | 0 (0.00) | 0 (0.00) |  |
|  |  |  |  |  |  |  |
| Earache |  |  |  |  |  |  |
| 0 | 467 (95.31) | 32 (47.06) | 77 (65.81) | 34 (70.83) | 161 (73.18) | <0.0001 |
| 1-3 | 23 (4.69) | 36 (52.94) | 38 (32.48) | 14 (29.16) | 58 (26.36) |  |
| 4 | 0 (0.00) | 0 (0.00) | 2 (1.71) | 0 (0.00) | 1 (0.45) |  |

*Note.* Only symptoms where at least one child from at least one of the PCC trajectory groups consistently reported it over follow-up are shown (cough, hoarse voice, abdominal pain, diarrhoea, welts, feet sores and other symptoms were not persistently reported for any PCC trajectory group so excluded from table; sleeping problems also excluded as only asked about at two time-points). * p-values are for Chi-Square tests/Fishers Exact (denoted by ^a^)

References

1.	Ministry of Housing Communities & Local Government. English indices of deprivation 2015 2015 [25 April 2023]. Available from: <https://www.gov.uk/government/statistics/english-indices-of-deprivation-2015>.

2.	Merzon E, Weiss MD, Cortese S, Rotem A, Schneider T, Craig SG, et al. The Association between ADHD and the Severity of COVID-19 Infection. J Atten Disord. 2022;26(4):491-501. Epub 20210402. doi: 10.1177/10870547211003659. PubMed PMID: 33797281.

3.	Merzon E, Weiss M, Krone B, Cohen S, Ilani G, Vinker S, et al. Clinical and Socio-Demographic Variables Associated with the Diagnosis of Long COVID Syndrome in Youth: A Population-Based Study. Int J Environ Res Public Health. 2022;19(10). Epub 20220515. doi: 10.3390/ijerph19105993. PubMed PMID: 35627530; PubMed Central PMCID: PMCPMC9141083.

4.	Wille N, Badia X, Bonsel G, Burström K, Cavrini G, Devlin N, et al. Development of the EQ-5D-Y: a child-friendly version of the EQ-5D. Qual Life Res. 2010;19(6):875-86. Epub 20100420. doi: 10.1007/s11136-010-9648-y. PubMed PMID: 20405245; PubMed Central PMCID: PMCPMC2892611.

5.	SDQ. Scoring the SDQ 2016 [26th July 2024]. Available from: <https://www.sdqinfo.org/py/sdqinfo/c0.py>.

6.	Warwick Medical School. Collect, score, analyse and interpret WEMWBS 2023 [20th November 2024]. Available from: <https://warwick.ac.uk/fac/sci/med/research/platform/wemwbs/using/howto/>.

7.	Stephenson T, Allin B, Nugawela MD, Rojas N, Dalrymple E, Pinto Pereira S, et al. Long COVID (post-COVID-19 condition) in children: a modified Delphi process. Archives of Disease in Childhood. 2022;107(7):674-80. doi: 10.1136/archdischild-2021-323624.
